# Discovery of natural few-layer graphene on the Moon

**DOI:** 10.1093/nsr/nwae211

**Published:** 2024-06-17

**Authors:** Wei Zhang, Qing Liang, Xiujuan Li, Lai-Peng Ma, Xinyang Li, Zhenzhen Zhao, Rui Zhang, Hongtao Cao, Zizhun Wang, Wenwen Li, Yanni Wang, Meiqi Liu, Nailin Yue, Hongyan Liu, Zhenyu Hu, Li Liu, Qiang Zhou, Fangfei Li, Weitao Zheng, Wencai Ren, Meng Zou

**Affiliations:** Key Laboratory of Automobile Materials Ministry of Education, School of Materials Science & Engineering, Electron Microscopy Center, International Center of Future Science, Jilin Provincial International Cooperation Key Laboratory of High-Efficiency Clean Energy Materials, Jilin University, China; Key Laboratory of Automobile Materials Ministry of Education, School of Materials Science & Engineering, Electron Microscopy Center, International Center of Future Science, Jilin Provincial International Cooperation Key Laboratory of High-Efficiency Clean Energy Materials, Jilin University, China; Key Laboratory of Bionic Engineering, Ministry of Education, Jilin University, China; Shenyang National Laboratory for Materials Science, Institute of Metal Research, Chinese Academy of Sciences, China; Synergetic Extreme Condition High-Pressure Science Center, State Key Laboratory of Superhard Materials, College of Physics, Jilin University, China; Key Laboratory of Automobile Materials Ministry of Education, School of Materials Science & Engineering, Electron Microscopy Center, International Center of Future Science, Jilin Provincial International Cooperation Key Laboratory of High-Efficiency Clean Energy Materials, Jilin University, China; Key Laboratory of Bionic Engineering, Ministry of Education, Jilin University, China; Key Laboratory of Bionic Engineering, Ministry of Education, Jilin University, China; Key Laboratory of Automobile Materials Ministry of Education, School of Materials Science & Engineering, Electron Microscopy Center, International Center of Future Science, Jilin Provincial International Cooperation Key Laboratory of High-Efficiency Clean Energy Materials, Jilin University, China; Key Laboratory of Automobile Materials Ministry of Education, School of Materials Science & Engineering, Electron Microscopy Center, International Center of Future Science, Jilin Provincial International Cooperation Key Laboratory of High-Efficiency Clean Energy Materials, Jilin University, China; Key Laboratory of Automobile Materials Ministry of Education, School of Materials Science & Engineering, Electron Microscopy Center, International Center of Future Science, Jilin Provincial International Cooperation Key Laboratory of High-Efficiency Clean Energy Materials, Jilin University, China; Key Laboratory of Automobile Materials Ministry of Education, School of Materials Science & Engineering, Electron Microscopy Center, International Center of Future Science, Jilin Provincial International Cooperation Key Laboratory of High-Efficiency Clean Energy Materials, Jilin University, China; Key Laboratory of Automobile Materials Ministry of Education, School of Materials Science & Engineering, Electron Microscopy Center, International Center of Future Science, Jilin Provincial International Cooperation Key Laboratory of High-Efficiency Clean Energy Materials, Jilin University, China; Key Laboratory of Automobile Materials Ministry of Education, School of Materials Science & Engineering, Electron Microscopy Center, International Center of Future Science, Jilin Provincial International Cooperation Key Laboratory of High-Efficiency Clean Energy Materials, Jilin University, China; Deep Space Exploration Lab, China; Deep Space Exploration Lab, China; Lunar Exploration and Space Engineering Center, China; Synergetic Extreme Condition High-Pressure Science Center, State Key Laboratory of Superhard Materials, College of Physics, Jilin University, China; Synergetic Extreme Condition High-Pressure Science Center, State Key Laboratory of Superhard Materials, College of Physics, Jilin University, China; Key Laboratory of Automobile Materials Ministry of Education, School of Materials Science & Engineering, Electron Microscopy Center, International Center of Future Science, Jilin Provincial International Cooperation Key Laboratory of High-Efficiency Clean Energy Materials, Jilin University, China; Shenyang National Laboratory for Materials Science, Institute of Metal Research, Chinese Academy of Sciences, China; Key Laboratory of Bionic Engineering, Ministry of Education, Jilin University, China

## Abstract

Natural few-layer graphene is unambiguously identified from the Chang'e-5 lunar soil samples, which serves as a new platform for investigating extraterrestrial bodies.

Carbon is a fundamental element for understanding the formation and evolution of planetary bodies. The origin of the Moon has aroused intensive interest and debate, from which several hypotheses have been proposed. The prevalent giant impact theory has been strongly supported by the notion of a carbon-depleted Moon that derived from the early analysis of Apollo samples. Recently, this consensus has been significantly challenged by the observation of global carbon ion fluxes from the Moon, suggesting the presence of indigenous carbon on the Moon [1]. This observation is consistent with the presence of graphite in the lunar soil [2]. Therefore, it is highly desirable to unravel the crystalline structure of the indigenous carbon phase by conducting further characterization studies on the young lunar samples.

Graphene has revolutionized the research of condensed matter physics and materials science with its novel physical phenomena and extraordinary properties [3]. It plays an increasingly important role in extensive areas including planetary and space science. It is estimated that ∼1.9% of total interstellar carbon is in the form of graphene [4] and protosolar graphene has been identified in the carbonaceous chondrites [5]. Since graphene has been routinely prepared by using artificial techniques with distinct morphologies and properties as determined by the specific formation process, the composition and structure characterization of natural graphene would provide rich information on the geologic evolution of parent bodies.

Analysis of soil samples is the most precise method available for determining the composition and structure of the Moon and investigating its geologic origins and histories. The Chang'e-5 (CE-5) lunar soils are the youngest mare basalt samples reported so far [6]. Recent studies have identified ilmenite (FeTiO_3_), pentlandite, amorphous features and even photosynthetic catalysts in CE-5 lunar soil (Supporting Information I). Further investigation may lead to important scientific discoveries in astronomy, geography, chemistry and materials science [7]. Herein, we report the discovery and direct microscopy visualization of natural few-layer graphene in the CE-5 lunar soil samples, by utilizing a variety of characterization techniques (Supporting Information II). Graphene is embedded as individual flakes or formed as part of a carbon shell enclosing the mineral particles. Our result reveals one typical structure of indigenous carbon in the Moon and its formation mechanism has been proposed. This finding may reinvent the understanding of chemical components, geography episodes and the history of the Moon.

The CE-5 lunar regolith was drilled at a depth of ∼0.25 m in the northern Oceanus Procellarum at 51.916° W longitude and 43.058° N latitude on the lunar frontal surface on 1 December 2020, which has not been heavily affected by human interference. Figure 1a shows the morphology of the CE-5 lunar soil sample investigated in this study. It features an olive-like shape of 2.856 mm in length and 1.592 mm in width. Its surface is irregular, with the height gradually increasing from left to right. By using the correlative scanning electron microscopy (SEM)/Raman technique [8], Raman spectra were then collected on several spots with relatively high carbon content (Areas A–D; Area E is a reference of a carbon-free location in Fig. 1b), as shown in Fig. 1c. The prominent G band at ∼1580 cm^−1^ is observed, suggesting that the carbon species is dominated by the sp^2^ hybrid structure. Meanwhile, the D band at 1330–1390 cm^−1^ is discernible, which is related to the defective sp^3^ hybrid carbon atoms. However, the relative intensity ratio *I*_D_/*I*_D+G_ (∼0.28) is low, indicating a relatively high crystalline quality of the graphitic carbon. The presence of the 2D band at ∼2674 cm^−1^ is also identified. SEM-energy dispersive X-ray spectroscopy (EDS) mapping reveals that the sample contains C, O, Na, Mg, Al, Si, Ca, Ti and Fe (Fig. S1), which is consistent with a mixture of several minerals. A careful comparison shows that the areas (A/B/C/D) with the presence of carbon contain Fe, whereas no Fe was detected for the one (E) free of carbon, suggesting that the Fe-containing minerals facilitate the graphitization. Meanwhile, scanning transmission electron microscopy (STEM)-EDS imaging indicates that C, O, Na, Mg, Al, Si, Ca, Ti, Fe and Mo are distributed in the sample (Fig. S2). Si and especially Ca are distributed in aggregates, which agrees with a mixture of silicon/calcium-based compounds. We also notice that Mo was not detected by using SEM-EDS (Fig. S1), which is attributed to its uneven distribution. The distribution of Fe varies from one area to another (EDS shown in Figs S1–S3). HRTEM analyses were performed to characterize the structure details. In the low-magnification bright-field image (Fig. 1d), the sample appears as a flake containing small nanoparticles. To clarify the valence states of Fe, EELS analysis was performed on the two spots marked in the STEM image. Both spots contain Fe, and the Fe L_3_ peak in Spot 1 shows higher energy than that in Spot 2. Further examination under high magnification confirms the graphitic carbon detected by Raman spectra to contain few-layer graphene (Fig. 1e1–e3). Both bilayer and multi-layer structures are observed with the interplanar spacing of 0.35 nm, consistently with the spacing of graphite (002). We notice that graphene is abundant near the metal-containing (including Fe) compound, as also described in the following core–shell structures. Its surface composition was further examined by using the highly sensitive time-of-flight secondary ion mass spectrometry (Fig. S4). N and S are identified, which were not detected by using SEM-EDS and STEM-EDS. Overall, Si, Al, Ca and O are dominating in CE-5 samples while C, N, Na, Mg, Ti, Fe, Mo and S are minor.

**Figure 1.**
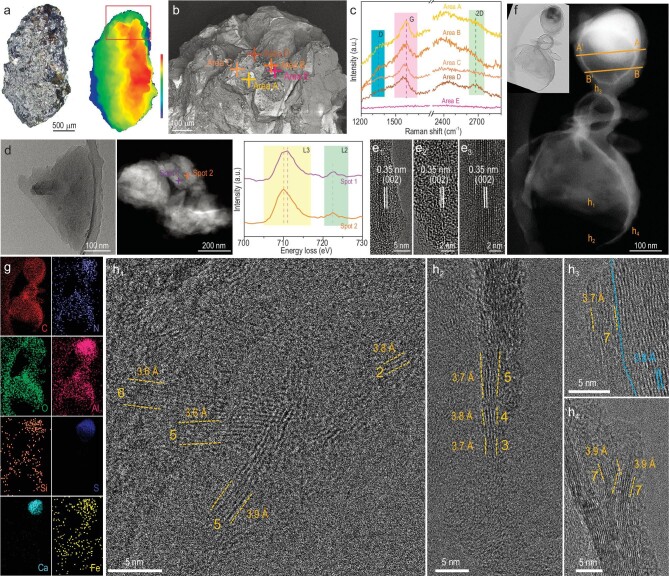
Structural and compositional characterization of few-layer graphene in the CE-5 lunar soil sample. (a) Laser scanning confocal microscopy image and height distribution. (b) Backscattered electron SEM image and (c) Raman spectra corresponding to different areas. (d) Representative low-magnification bright-field transmission electron microscopy (TEM) image, Cs-corrected high-angle annular dark-field (HAADF)-STEM image and the corresponding electron energy loss spectroscopy (EELS) Fe L-edge spectra for different areas. (e1–e3) Cs-corrected high-resolution TEM (HRTEM) images. (f) HAADF-STEM image of a core–shell structure with an abundance of few-layer graphene (upper left inset: bright-field TEM image of corresponding area), (g) EDS elemental maps showing spatial distributions of the elements and (h) Cs-corrected HRTEM images of (h1–h4) in the corresponding areas marked in (f). Both layer number and average interplanar spacing of few-layer graphene are labeled.

In addition to discrete flakes, few-layer graphene is also formed in a core–shell structure (Fig. 1f). A thin carbon shell encloses a core of complicated compounds with similar constituting elements to those aforementioned regions. C, N, O, Al, Si, S, Ca and Fe are distributed throughout the structure and chemical fluctuation exists for some elements, particularly for Ca, Al and S (Fig. 1g). Along line A–A′ (Fig. S5), the distribution intensity of C is high at both ends of the line and low in the middle, indicating that carbon species is mainly distributed in the shell. Conversely, the signals of Al, Ca and S are concentrated in the middle and barely visible on the two ends, indicating that Al, Ca and S are mainly present in the core. Along line B–B′, the distribution intensity of C is higher on both ends and lower in the middle, which is the same as line A–A′, indicating that carbon species is mainly in the shell. Al and S species show a similar distribution yet with much lower intensity. HRTEM was then used to analyse the structure of the carbon shell. Figure 1h1–h4 shows the HRTEM images of the corresponding h1–h4 areas in Fig. 1f. At areas h1–h3, few-layer graphene (two to seven layers) is observed with a slightly disordered fringe. Moreover, the lattice spacing of (002) is 0.36–0.39 nm, which is 3%–15% larger than the nominal 0.34 nm. Their results agree with the graphene with few defects as revealed by the Raman spectra. Graphene and thin graphite with higher crystalline quality are found together with metal-containing (e.g. Fe) compounds at area h4, indicating that the latter promotes graphitization. This result verifies the above observation of high-quality graphene formed with Fe-bearing compounds.

The identification of graphene in the core–shell structure suggests a bottom-up synthesis process rather than exfoliation, which generally involves a high-temperature catalytic reaction. Therefore, a formation mechanism of few-layer graphene and graphitic carbon is proposed here. Volcanic eruption, a typical high-temperature process, occurred on the Moon [9]. Lunar soil can be stirred up by solar wind and high-temperature plasma discharge can be generated on the Moon's surface. As illustrated by Fig. S6, the Fe-bearing mineral particles, such as olivine and pyroxene, in lunar soil might catalyse the conversion of carbon-containing gas molecules in the solar wind or polycyclic aromatic hydrocarbons into graphitic carbon of different thicknesses and morphologies on their surfaces [5,10], including few-layer graphene flakes (Fig. 1e1–e3) and carbon shells (Fig. 1h1–h4). The blue line is referred to graphitic carbon. In short, the formation of graphitic carbon may be attributed to high-temperature processes resulting from volcanic eruptions. Importantly, this mechanism suggests the presence of a carbon-capture process on the Moon, which might have led to the gradual accumulation of indigenous carbon. Since the discovery of graphene in meteorites [5] or on the Moon is extremely rare, impact processes from meteorites, which create high-temperature and high-pressure environments, may also lead to the formation of few-layer graphene and graphitic carbon. Further research is needed to understand its formation mechanism in detail.

In lunar soil samples, we identified few-layer graphene formed together with complex minerals. This is the first study to verify the presence of natural few-layer graphene in lunar soil samples by examining its microstructure and composition. Our finding provides new insights into the origin of the Moon, supporting the hypothesis of a carbon-containing Moon. Moreover, the exotic properties of graphene are highly structurally and environmentally dependent [3]. Further in-depth property investigation of natural graphene would provide more information on the geologic evolution of the Moon. In turn, the mineral-catalysed formation of natural graphene sheds light on the development of low-cost scalable synthesis techniques for high-quality graphene. Therefore, a new lunar exploration program may be promoted and some forthcoming breakthroughs can be expected.

## Supplementary Material

nwae211_Supplemental_File

## References

[1] Yokota S, Terada K, Saito Y et al. Sci Adv 2020; 6: eaba1050. 10.1126/sciadv.aba1050 32494721 PMC7202878

[2] Steele A, McCubbin F, Fries M et al. Science 2010; 329: 51. 10.1126/science.1190541 20595608

[3] Geim AK, Novoselov KS. Nat Mater 2007; 6: 183–91. 10.1038/nmat1849 17330084

[4] Chen XH, Li A, Zhang K. Astrophys J 2017; 850: 104. 10.3847/1538-4357/aa93d5

[5] Giri C, Steele A, Fries M. Planet Space Sci 2021; 203: 105267. 10.1016/j.pss.2021.105267

[6] Li Q, Zhou Q, Liu Y et al. Nature 2021; 600: 54–8. 10.1038/s41586-021-04100-2 34666338 PMC8636262

[7] Tian H, Wang H, Chen Y et al. Nature 2021; 600: 59–63. 10.1038/s41586-021-04119-5 34666339 PMC8636255

[8] Liu FX, Zou X, Yue NL et al. Cell Rep Phys Sci 2023; 4: 101607. 10.1016/j.xcrp.2023.101607

[9] Cao H, Wang C, Chen J et al. Geophys Res Lett 2022; 49: e2022GL099282. 10.1029/2022GL099282

[10] Shavelkina M, Amirov R, Bilera I. Mater Today: Proc 2018; 5: 25956–61.

