# Supplementary material for: Discovery of natural few-layer graphene on the Moon
Source: Natl Sci Rev. 2024 Jun 17;11(12):nwae211. doi: 10.1093/nsr/nwae211 (PMC11660913; doi:10.1093/nsr/nwae211)
Supplement: nwae211_Supplemental_File [file nwae211_supplemental_file.pdf]

Supporting information

Discovery of Natural Few-Layer Graphene on the Moon

Wei Zhang[1,*], Qing Liang[1], Xiujuan Li[2,*], Lai-Peng Ma[3], Xinyang Li[4], Zhenzhen Zhao[1], Rui Zhang[2], Hongtao Cao[2], Zizhun Wang[1], Wenwen Li[1], Yanni Wang[1], Meiqi Liu[1], Nailin Yue[1], Hongyan Liu[1], Zhenyu Hu[5], Li Liu[5,6], Qiang Zhou[4], Fangfei Li[4], Weitao Zheng[1], Wencai Ren[3,*] and Meng Zou[2,*]

[1]Key Laboratory of Automobile Materials Ministry of Education, School of Materials Science & Engineering, Electron Microscopy Center, International Center of Future Science, Jilin Provincial International Cooperation Key Laboratory of High-Efficiency Clean Energy Materials, Jilin University, Changchun 130012, China
[2]Key Laboratory of Bionic Engineering, Ministry of Education, Jilin University, Changchun 130025, China
[3]Shenyang National Laboratory for Materials Science, Institute of Metal Research, Chinese Academy of Sciences, Shenyang 110016, China
[4]Synergetic Extreme Condition High-Pressure Science Center, State Key Laboratory of Superhard Materials, College of Physics, Jilin University, Changchun 130012, China
[5]Deep Space Exploration Lab, Beijing 100195, China
[6]Lunar Exploration and Space Engineering Center, Beijing 100195, China

*Corresponding authors. Emails: weizhang@jlu.edu.cn (W.Z.); xiujuanli@jlu.edu.cn (X.J.L.); wcren@imr.ac.cn (W.C.R.); zoumeng@jlu.edu.cn (M.Z.)

I. Some background for the CE-5 sample

Recent studies have identified Basalt (*1*), ilmenite ($FeTiO_3$) (*2*), pentlandite (*3*), amorphous features (*4*), and even photosynthetic catalysts(*5*) in CE-5 lunar soil.

(1) H. Cao, C. Wang, J. Chen, X. Che, X. Fu, Y. Shi, D. Liu, Z. Ling, L. Qiao, X. Lu, Xiaobin Qi, C. Yin, P. Liu, C. Liu, Y. Xin, J. Liu, A Raman spectroscopic and microimage analysis perspective of the Chang'e-5 lunar samples. *Geophys. Res. Lett.* **49**, e2022GL099282 (2022).

(2) J.-H. Li, Non-destructive identification and quantification of ilmenite from a single particle of the Chang'e-5 lunar soil sample. *At. Spectrosc.* **43**, 283-290 (2022).

(3) X. Tang, H. Tian, S. Sun, L. Gu, Q. Li, X. Li, J. Li, Origin and implication of pentlandite in Chang'e-5 lunar soils. *Lithos* **458-459**, 107342 (2023).

(4) J. Xi, L. Ma, H. Xian, G. Wang, J. Xing, J. Wei, J. Zhu, H. He, In situ micro-XRD methods for identifying glass and minerals in extraterrestrial samples. *At. Spectrosc.* **43**, 19-27 (2022).

(5) Y. Yao, L. Wang, X. Zhu, W. Tu, Y. Zhou, R. Liu, J. Sun, B. Tao, C. Wang, X. Yu, L. Gao, Y. Cao, B. Wang, Z. Li, W. Yao, Y. Xiong, M. Yang, W. Wang, Z. Zou, Extraterrestrial photosynthesis by Chang'E-5 lunar soil. *Joule* **6**, 1008-1014 (2022).

II. Materials and Methods

Abbreviation of Terminology

SEM: scanning electron microscopy
TEM: transmission electron microscopy
HRTEM: high-resolution transmission electron microscopy
HAADF: high-angle annular dark-field
STEM: scanning transmission electron microscopy
EDS: energy dispersive X-ray spectroscopy
EELS: electron energy loss spectroscopy
TOF-SIMS: time-of-flight secondary ion mass spectrometry

Laser scanning confocal microscopy (LSCM) image was performed on an OLS 5100 LSCM. TESCAN All-in-One System (TESCAN S9000G) was used to obtain the information via SEM (including secondary-electron and backscattered electron) images, EDS (Oxford Instruments), Raman spectra (WITec alpha300 R) and the TOF-SIMS result. GeminiSEM 360 was employed to analyze the morphology.

TEM and EDS were obtained with a TEM (JEM-2100F). HAADF-STEM, HRTEM, and EELS characterizations were performed on a spherical aberration (Cs) corrected TEM (JEM ARM300F Grand ARM), with an accelerating voltage of 300 kV. The low-order aberrations have been tuned to an acceptable level that A1 is <2 nm, Cs is <0.5 μm, A2 is <10 nm. The TEM samples were acquired by scraping the lunar sample, followed by the deposition process on a holey carbon film for microscopy observation. Since electron beam radiation damage is unfavorable for the analysis of the original structure of samples in TEM characterization, Fischione's Cryo-holder 2550 was employed to reduce the radiation damage as much as possible.

Supplementary figures

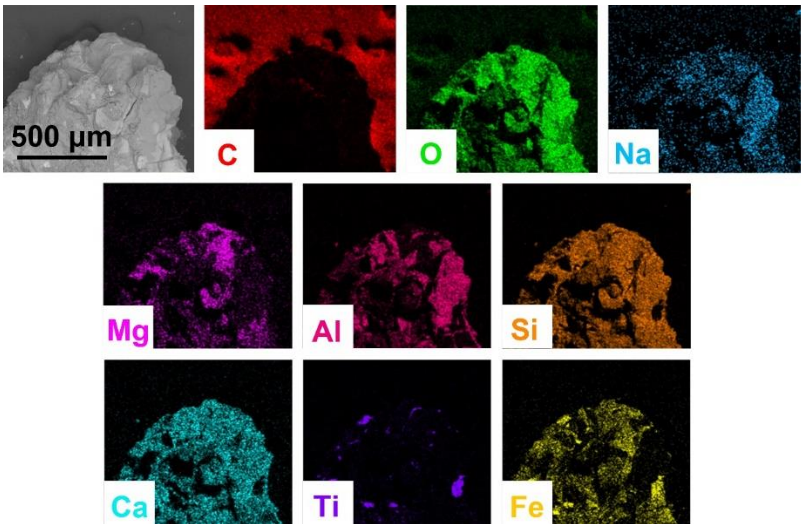

Figure S1 SEM image and the corresponding EDS elemental mapping analysis of the CE-5 lunar soil sample.

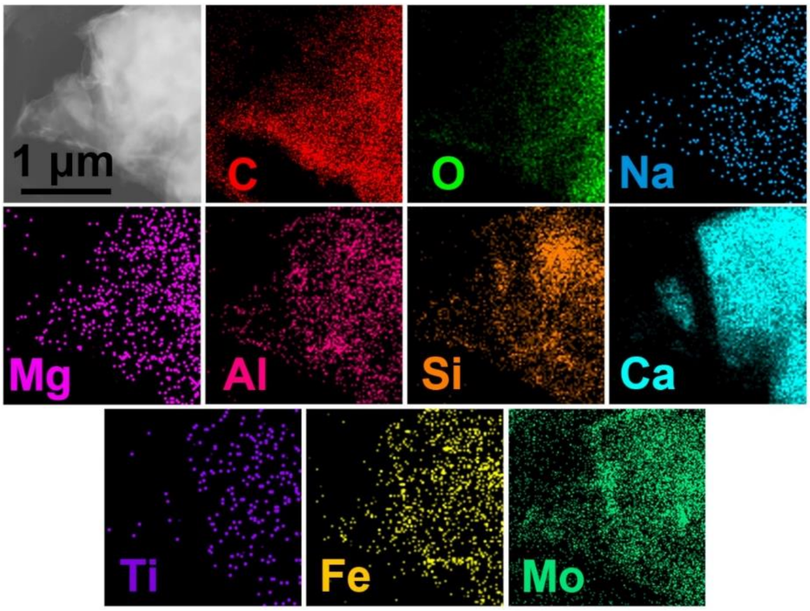

Figure S2 STEM image and the corresponding EDS elemental mapping analysis of the CE-5 lunar soil sample.

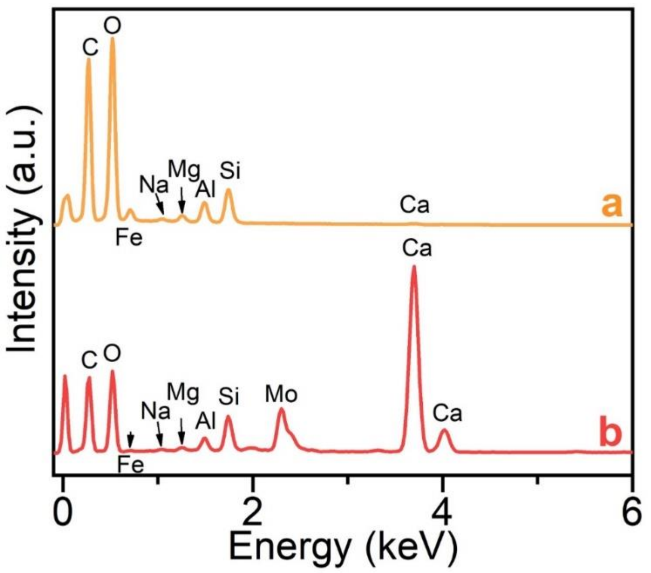

Figure S3 EDS spectra of different areas in (a, Fig. S1) and (b, Fig. S2) of the CE-5 lunar soil sample.

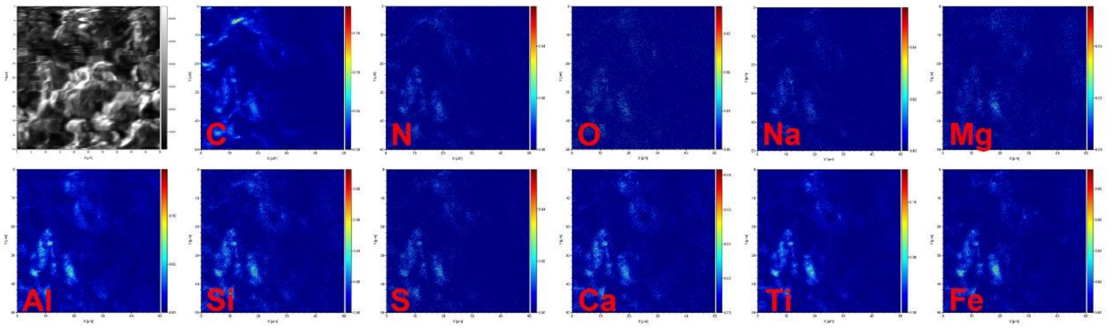

Figure S4 Correlative SEM/TOF-SIMS (time-of-flight secondary ion mass spectrometry) result of the CE-5 lunar soil sample for the distribution of elements.

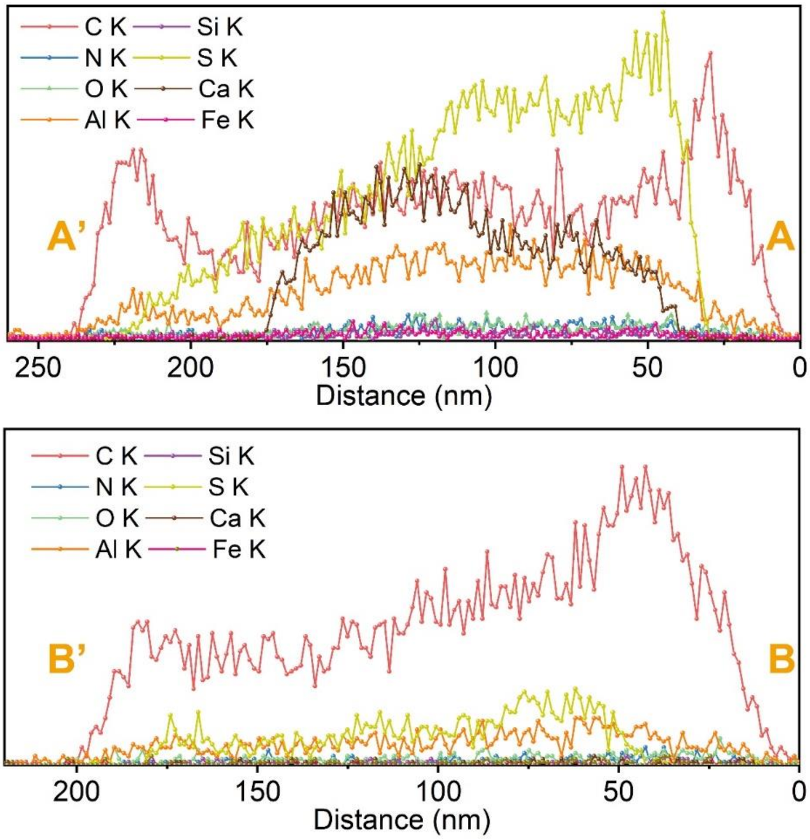

Figure S5 EDS line profiles of the core-shell structure, showing fluctuated spatial distribution of the elements.

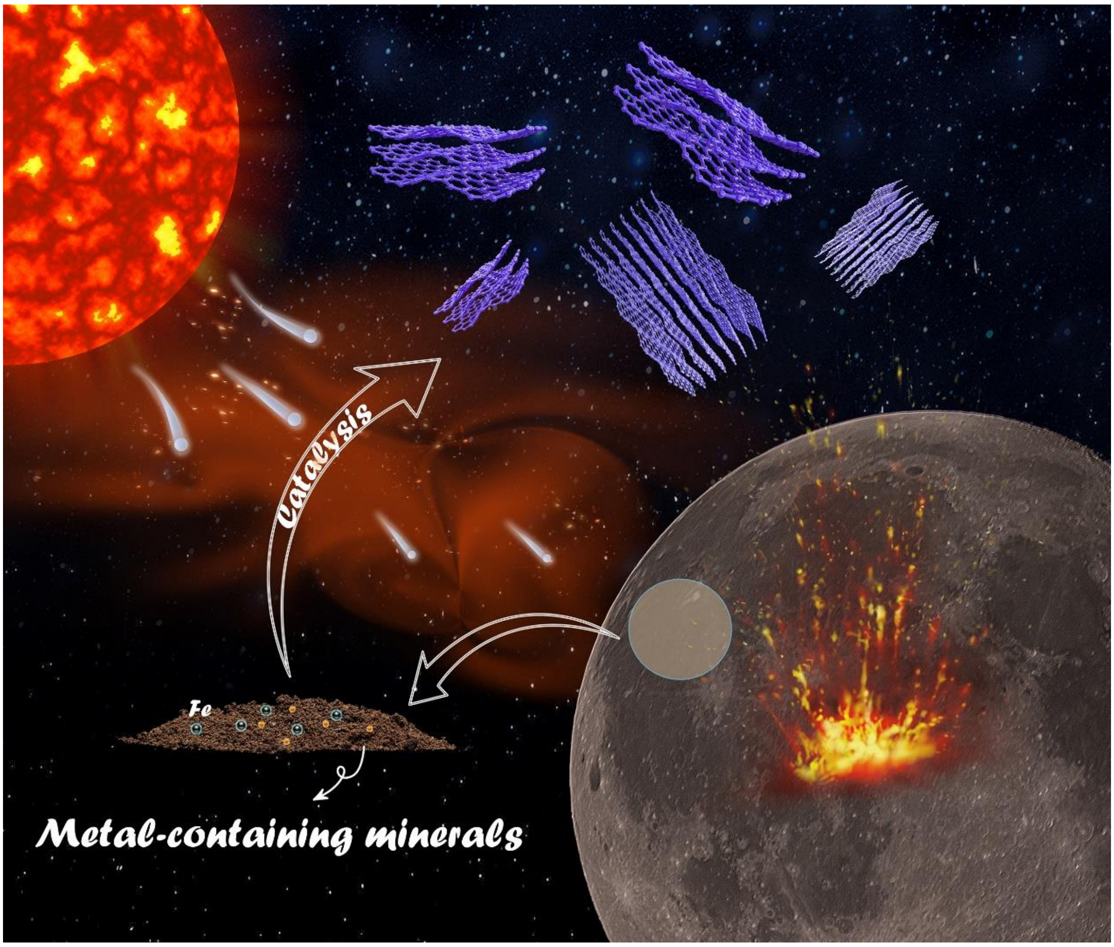

Figure S6 Illustration of the possible formation process of graphene on the Moon.
